# The Characteristic Changes in Hepatitis B Virus X Region for Hepatocellular Carcinoma: A Comprehensive Analysis Based on Global Data

**DOI:** 10.1371/journal.pone.0125555

**Published:** 2015-05-05

**Authors:** Wenwen Li, Kaku Goto, Yasuo Matsubara, Sayaka Ito, Ryosuke Muroyama, Qiang Li, Naoya Kato

**Affiliations:** 1 Division of advanced genome medicine, Advanced clinical research center, The Institute of Medical Science, The University of Tokyo, Tokyo, Japan; 2 Affiliated Infectious Disease Hospital of Shandong University, Jinan, Shandong, China; Chiba University, Graduate School of Medicine, JAPAN

## Abstract

**Objectives:**

Mutations in hepatitis B virus (HBV) X region (HBx) play important roles in hepatocarcinogenesis while the results remain controversial. We sought to clarify potential hepatocellular carcinoma (HCC)-characteristic mutations in HBx from HBV genotype C-infected patients and the distribution of those mutations in different disease phases and genotypes.

**Methods:**

HBx sequences downloaded from an online global HBV database were screened and then classified into Non-HCC or HCC group by diagnosis information. Patients' data of patient age, gender, country or area, and viral genotype were also extracted. Logistic regression was performed to evaluate the effects of mutations on HCC risk.

**Results:**

1) Full length HBx sequences (HCC: 161; Non-HCC: 954) originated from 1115 human sera across 29 countries/areas were extracted from the downloaded 5956 HBx sequences. Genotype C occupied 40.6% of Non-HCC (387/954) and 89.4% of HCC (144/161). 2) Sixteen nucleotide positions showed significantly different distributions between genotype C HCC and Non-HCC groups. 3) Logistic regression showed that mutations A1383C (OR: 2.32, 95% CI: 1.34-4.01), R1479C/T (OR: 1.96, 95% CI: 1.05-3.64; OR: 5.15, 95% CI: 2.53-10.48), C1485T (OR: 2.40, 95% CI: 1.41-4.08), C1631T (OR: 4.09, 95% CI: 1.41-11.85), C1653T (OR: 2.58, 95% CI: 1.59-4.19), G1719T (OR: 2.11, 95% CI: 1.19-3.73), and T1800C (OR: 23.59, 95% CI: 2.25-247.65) were independent risk factors for genotype C HBV-related HCC, presenting different trends among individual disease phases. 4) Several genotype C HCC risk mutations pre-existed, even as major types, in early disease phases with other genotypes.

**Conclusions:**

Mutations associated with HCC risk were mainly located in HBx transactivation domain, viral promoter, protein/miRNA binding sites, and the area for immune epitopes. Furthermore, the signatures of these mutations were unique to disease phases leading to HCC, suggesting molecular counteractions between the virus and host during hepatocarcinogenesis.

## Introduction

Chronic hepatitis B virus (HBV) infection remains a major health problem with more than 400 million people infected worldwide. Persistent HBV infection induces advanced liver diseases including liver cirrhosis (LC) and hepatocellular carcinoma (HCC). It accounts for approximately 50% of the HCC cases worldwide and even 80–90% in the area where HBV is highly prevalent [1]. The progression from asymptomatic HBV carrier (ASC) to HCC usually takes 20–30 years, while patients’ outcomes varied a lot according to the balance between host immune system and the virus.

The HBV genome is a 3.2 kb long and partially double-stranded circular DNA. It contains four overlapped open reading frames (ORFs): P, C, S, and X, encoding polymerase, core and HBe antigen, surface antigen, and HBx protein, respectively. Transcription of these proteins is controlled under four promoters, preS1, preS2, core and X, and two enhancers, Enh 1 and Enh 2, in the viral genome, which overlap with those ORFs. Due to the lack of proof-reading ability of the viral reverse transcriptase, HBV genome has a higher mutation ratio than other DNA viruses, approximately 1.4–3.2×10^-5^ nucleotide (nt) mutations per site per year. Moreover, the endogenous and exogenous pressures, exemplified by host immunity and intervention by antiviral drugs and vaccines, respectively, make the virus mutations more complex, thereby forming various HBV genotypes.

Based on the difference more than 8% of whole sequences, HBV genome is divided into at least 10 genotypes (A-J). Different genotypes have distinct geographical distributions and usually induce various clinical outcomes. For instance, genotype C, the most prevalent genotype in Asia, had shown to be more associated with HCC than genotype B [2,3]. Genotype D prevalent in Africa, Europe, the Mediterranean region and India and genotype F prevalent in Central and South America were also proved to be more carcinogenetic strains in local cohorts [4]. Accumulating data had shown that certain HBx mutations in specific HBV strains are critical for severe liver diseases including HCC [5–9]. However, the results remained inconsistent even in the same genotypes or cohorts with similar ethnic background. One possible reason may be the limited nucleotides/patients and cohorts investigated. Currently, it is still obscure if there were universal mutations responsible for HCC and if HCC risk mutations exist only in certain genotypes. Here through a global HBV database, we explored the characteristic mutations in genotype C HBx for HCC and examined their distribution among different disease phases and different HBV genotypes.

## Materials and Methods

### Sequences collection

HBx sequences including both nucleotide and amino acid sequences were downloaded from Hepatitis Virus Database (HVDB, http://s2as02.genes.nig.ac.jp/) [10], a user-friendly online public nucleotide database focused on hepatitis viruses especially HBV and HCV. All hepatitis virus sequences deposited in HVDB were retrieved from DNA Data Bank of Japan (DDBJ) and all of the information was updated from DDBJ periodically. The version we exploited was DDBJ Rel. 95 in Dec 2013, which contained 5956 nucleotide sequences of HBV X region. All the formats of sequences were in conformity to Genbank.

### Sequences screening

#### Sequences multi-alignment

Sequences multi-alignment was firstly performed using ClustalW online analysis (DDBJ, http://ddbj.sakura.ne.jp/searches-e.html), results were examined manually twice.

#### Sequences exclusion criteria

We screened the sequences by attached information in HVDB database and sequence related publications in Pubmed (http://www.ncbi.nlm.nih.gov/pubmed) successively: publication, origin, diagnosis etc. Briefly, we set exclusion criteria as 1) since the attached information of database sequences were very limited, it’s impossible to distinguish the origin of all the sequences and the diagnosis of patients with HBV infection when publications concerned are unavailable. Therefore sequences with no any published information were excluded first; 2) since virus sequences from different origins may varied a lot, we next excluded sequences from Non-human origins and those from liver tissue or cell lines; 3) Sequences will be excluded if the related paper did not specify the diagnosis clearly. For instance, if a paper only stated that their sera samples were from patients with chronic HBV infection, all related sequences will be excluded; 4) When more than one sequence were from same patient, either from same or different time point, only one sequence with available information would be used; 5) Sequences from patients with acute disease phases, co-infection with other viruses, or complications were excluded; 6) Sequences with recombinant HBV genotypes were excluded; 7) Sequences with insertions or deletions were excluded.

### Sequence inclusion criteria

**Fig 1:**
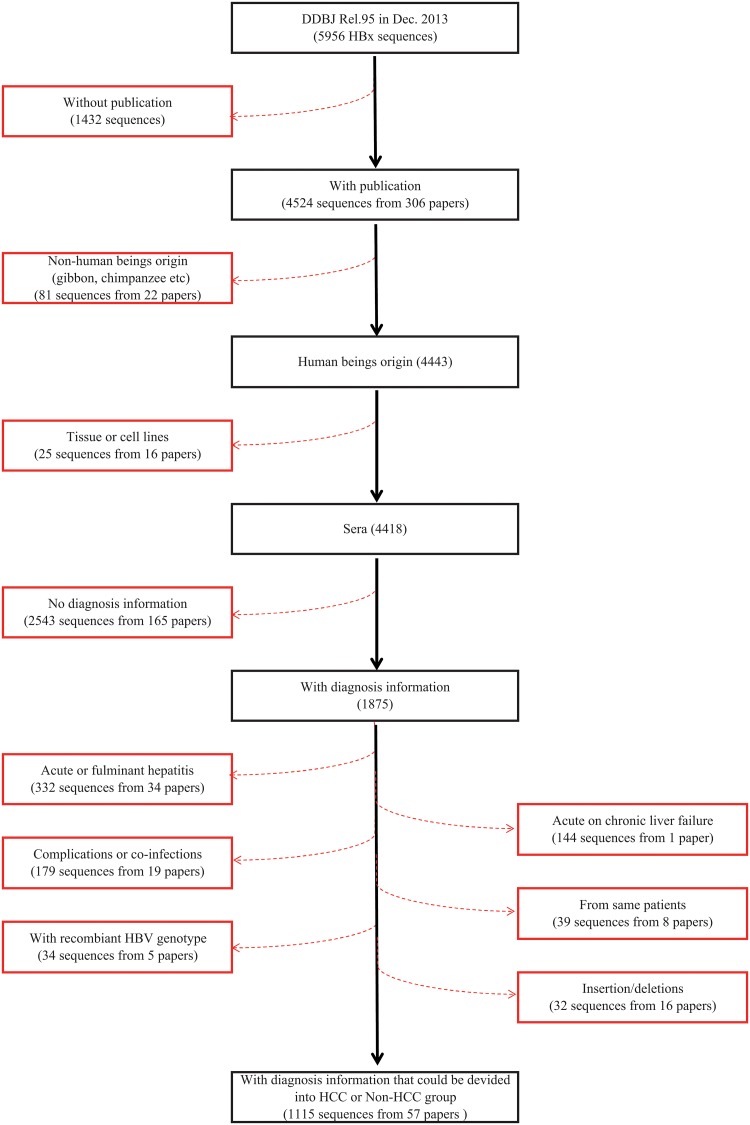
Flowchart for screening HBx sequences downloaded from an online global database. We downloaded HBx sequences from Hepatitis Virus Database (HVDB, http://s2as02.genes.nig.ac.jp/). The version we exploited was DDBJ Rel. 95, containing 5956 HBx sequences in total. Sequences were then screened successively by attached information such as publication, origin, and diagnosis. Sequences enrolled should be 1) Full length HBV X sequence; 2) human sera origin; and 3) with diagnosis information and thus could be classified to non-HCC or HCC group. Finally 1115 full length HBx sequences (HCC, 161; and Non-HCC, 954) from 57 publications were extracted for further analyses.

Sequences finally enrolled should be: 1) Full length HBV X sequence (465 bp, started from 1 at A of ATG and ended with 465 at A of TGA/TAA, or G of TAG); 2) human sera origin; and 3) with diagnosis information and thus could be classified to non-HCC or HCC group (Fig 1 and S1 Table). We screened the database, and extracted the information including patient age, gender, country or area, and viral genotype from the dataset, referring, as necessary, to the publications listed therein (S2 Table). Viral genotypes were also confirmed by online NCBI genotyping tool (http://www.ncbi.nlm.nih.gov/projects/genotyping/formpage.cgi).

### Sequences analyses

Continuous data were expressed as Mean (range) and were compared by t-test. Categorical data were analyzed by Fisher’s exact test (SPSS 16.0). *P* < 0.05 was considered to have significant differences. Logistic regression was performed to evaluate the effects of mutations on HCC risk. We set nucleotide residues which occupied lower ratios in Non-HCC sequences and higher ratios in HCC sequences as “Mutant” type. For instance, at nt1479 frequencies of the nucleotides C and T were increased in HCC compared with Non-HCC, and on the other hand, those for A and G were decreased, leading to the definition of 1479Y (C/T) as mutations.

### Ethics Statement

The need for ethical approval was waived by [Ethical Review Board, The Institute of Medical Science, The University of Tokyo] because the authors analyzed anonymized Hepatitis B Virus (HBV) sequences acquired from the Hepatitis Virus Database (HVDB, http://s2as02.genes.nig.ac.jp/), an online, public database. The information was anonymized and de-identified prior to the author's access of the data.

## Results

### General information of enrolled HBx sequences

One thousand and one hundred fifteen HBx sequences (HCC, 161; and Non-HCC, 954) covering 29 countries/areas were finally extracted from the downloaded 5956 HBV X sequences (Table 1). Non-HCC group included five types of patients. According to the publications exploited, patients with diagnosis information of AsC, inactive carriers (IC), CHB, or LC were considered to be Non-HCC. In addition, patients who got HBV related chronic liver diseases without HCC and lacked the information on cirrhosis status were enrolled into Non-HCC group as well. Genotype C occupied 40.6% of Non-HCC (387/954) and 89.4% of HCC (144/161). Genotype B, D, and E occupied 52.7% of Non-HCC sequences. According to the information available, we compared the patients’ age and gender between two groups. HCC group showed an average age 10 years older than that of Non-HCC group (*P* < 0.01) and male gender occupied higher ratio in HCC (42/45, 93.3%) than that of Non-HCC group (99/128, 77.3%) (*P* = 0.02). In Table 2, the demographic information of the enrolled 531 genotype C sequences was summarized. The majority of both genotype C Non-HCC group (79.8%) and HCC group (97.9%) were constituted of sequences from Japan, Mainland China and South Korea.

**Table 1:** The demographic information of enrolled sequences.

Group		HCC	Non-HCC	*P* value^a^
Number		161	954	
Gender	Known (M/F)	45 (42/3)	128 (99/29)	0.02
Age	Mean (n/range)	56.3 (46/26–89.8)	46.0 (130/5–86)	<0.01
Diagnosis	HCC	161 (100%)	/	/
	ASC	/	366 (38.4%)	/
	IC	/	27 (2.8%)	/
	CHB	/	118 (12.4%)	/
	LC	/	71 (7.4%)	/
	Unclear but no HCC	/	372 (39.0%)	/
Countries/Areas	Australia	1/0.6%	3/0.3%	/
(n/%)	Belgium	/	20/2.1%	/
	Bolivia	/	7/0.7%	/
	Brazil	/	1/0.1%	/
	Cameroon	/	5/0.5%	/
	Chile	/	21/2.2%	/
	France	/	1/0.1%	/
	Ghana	/	14/1.5%	/
	Guinea	/	78/8.2%	/
	Hong Kong	2/1.2%	47/4.9%	/
	India	/	53/5.6%	/
	Indonesia	2/1.2%	2/0.2%	/
	Iran	/	76/8.0%	/
	Ireland	/	1/0.1%	/
	Japan	108/67.1%	105/11.0%	/
	Mainland China	20/12.4%	239/25.1%	/
	Malaysia	/	30/3.1%	/
	Niger	/	18/1.9%	/
	Nigeria	/	47/4.9%	/
	Philippines	5/3.1%	9/0.9%	/
	Serbia	/	5/0.5%	/
	South Africa	/	17/1.8%	/
	South Korea	23/14.3%	40/4.2%	/
	Spain	/	1/0.1%	/
	Taiwan	/	7/0.7%	/
	Thailand	/	1/0.1%	/
	Turkey	/	100/10.5%	/
	Uzbekistan	/	5/0.5%	/
	Vietnam	/	1/0.1%	/
Genotypes	A	2	34	/
	B	14	76	/
	C	144	387	/
	D	/	268	/
	E	/	159	/
	F	/	27	/
	H	/	2	/
	I		1	/
	J	1	/	/

^a^Differences as proportions have been presented where calculable. HCC, hepatocellular carcinoma; ASC, asymptomatic HBV carriers; IC, inactive HBV carriers; CHB, chronic hepatitis B; LC, liver cirrhosis.

**Table 2:** The demographic information of enrolled genotype C sequences.

Group		HCC	Non-HCC	*P* value^a^
Number		144	387	
Gender	Known (M/F)	42 (39/3)	61 (52/9)	0.35
Age	Mean (n/range)	56.9 (43/35.9–89.8)	48.6 (61/5–74.3)	<0.01
Diagnosis	HCC	144 (100%)	/	/
	AsC	/	18 (4.7%)	/
	IC	/	/	/
	CHB	/	38(9.8%)	/
	LC	/	27 (7.0%)	/
	Unclear but no HCC	/	304 (78.6%)	/
Countries/Areas	Australia	1/0.7%	1/0.3%	/
	Belgium	/	1/0.3%	/
	Bolivia	/	1/0.3%	/
	Brazil	/	1/0.3%	/
	Cameroon	/	/	/
	Chile	/	/	/
	France	/	/	/
	Ghana	/	/	/
	Guinea	/	/	/
	Hong Kong	/	45/11.6%	/
	India	/	/	/
	Indonesia	/	1/0.3%	/
	Iran	/	/	/
	Ireland	/	/	/
	Japan	106/73.6%	85/22.0%	/
	Mainland China	12/8.3%	184/47.5%	/
	Malaysia	/	13/3.4%	/
	Niger	/	/	/
	Nigeria	/	/	/
	Philippines	2/1.4%	7/1.8%	/
	Serbia	/	/	/
	South Africa	/	/	/
	South Korea	23/16.0%	40/10.3%	/
	Spain	/	/	/
	Taiwan	/	4/1.0%	/
	Thailand	/	1/0.3%	/
	Turkey	/	1/0.3%	/
	Uzbekistan	/	1/0.3%	/
	Vietnam	/	1/0.3%	/

^a^Differences as proportions have been presented where calculable. HCC, hepatocellular carcinoma; AsC, asymptomatic HBV carriers; IC, inactive HBV carriers; CHB, chronic hepatitis B; LC, liver cirrhosis.

### Different nucleotide distribution between genotype C Non-HCC and HCC

We next examined each nucleotide position of genotype C HBx sequences between HCC and Non-HCC (Table 2). Sixteen out of all 465 positions showed significant differences (Table 3). In overlapped cis-elements, four were also included in Enh2 region (nt1636-1744), nine in core promoter (CP) region (nt1613-1849). Five positions in HCC group (A1383C, R1479Y, C1485T, C1653T, and G1719T) showed elevating mutant ratios more than 10% toward Non-HCC group and A1383C single mutation showed a relatively low mutant ratio (26%) in genotype C Non-HCC but elevated to more than 50% in genotype C HCC group.

**Table 3:** Sixteen significant differences of HBx nucleotide sequences between HBV genotype C infected patients with and without HCC.

Nucleotide changes	Nucleotide location in X region	Ratio in groups^a^		*P* value
		Non-HCC	HCC	
A → C	nt 1383	**25.8%**	**52.8%**	< 0.001
C → T	nt 1425	10.1%	17.4%	0.009
T → C	nt 1458	95.9%	100.0%	0.009
R → Y	nt 1479	**30.5%**	**64.6%**	< 0.001
C → T	nt 1485	**16.8%**	**29.9%**	0.003
G → A	nt 1511	7.0%	13.9%	0.028
G → A	nt 1569	0%	0.7%	0.018
G → A	nt 1630	81.4%	91.0%	0.012
C → T	nt 1631	1.8%	8.3%	0.001
C → T	nt 1653	**18.6%**	**35.4%**	< 0.001
A → T	nt 1689	0.5%	2.8%	0.049
G → T	nt 1719	**57.6%**	**82.6%**	< 0.001
A → G	nt 1721	85.3%	95.1%	0.002
A → G	nt 1757	94.6%	100.0%	0.005
G → A	nt 1775	86.3%	95.8%	<0.001
T → C	nt 1800	0.3%	3.5%	0.007

^a^Positions that changed more than 10% were marked in bold.

### Risky mutations of genotype C HBV related to HCC

We enrolled all the 16 point mutations into multivariate analyses for exploring the risky mutations for genotype C HBV in relation to HCC. Logistic regression showed that mutations A1383C (OR: 2.32, 95% CI: 1.34–4.01), R1479C/T (OR: 1.96, 95% CI: 1.05–3.64; OR: 5.15, 95% CI: 2.53–10.48), C1485T (OR: 2.40, 95% CI: 1.41–4.08), C1631T (OR: 4.09, 95% CI: 1.41–11.85), C1653T (OR: 2.58, 95% CI: 1.59–4.19), G1719T (OR: 2.11, 95% CI: 1.19–3.73), and T1800C (OR: 23.59, 95% CI: 2.25–247.65) were independent risk factors for genotype C HBV-related HCC (Table 4), where Stepwise Forward (Conditional) method for logistic regression was used and those variates with insignificant p values (*P* > 0.05) were not analyzed further for OR calculation. Corresponding amino acid (AA) sequences were also examined to find possible substitutions induced by those nucleotide mutations. We found six nonsynonymous (aa36, Thr → Pro; aa36, Thr → Ser; aa38, Pro → Ser; aa94, His → Tyr; aa116, Val → Leu; and aa143, Cys → Arg) and two synonymous substitutions (aa4, Arg → Arg; aa86, His → His) in HBx and three synonymous substitutions in the overlapped polymerase (aa764, Leu → Leu; aa796, Gly → Gly; aa798, Tyr → Tyr). The comparison of the whole AA sequences between HCC and Non-HCC exhibited similar results. Among 154 aa positions, differently distributed between the two groups in multivariate analysis were the nonsynonymous mutations above aa36, 38, 94, 116 and 143 (S3 Table).

**Table 4:** Seven nucleotide mutations of HBx sequences were independent risk factors for genotype C HBV-related **HCC**.

Mutations^a^	Nucleotide location		AA location		Mutant ratio in groups (%)		OR	*P*
	X	Cis-elements	HBx	Polymerase	Non-HCC	HCC	(95% CI)	value
**A**GG → **C**GG	nt1383	miRNA binding site	4	764	25.8	52.8	2.32	0.003
			(Arg, Arg)	(Leu, Leu)			(1.34, 4.01)	
**C**TA → **T**TA	nt1425							0.275
**T**CC → **C**CC	nt1458							0.078
**R**CT → **C**CT	nt1479	B cell epitope	36	796	22.7	49.3	1.96	0.034
			(Thr, Pro)	(Gly, Gly)			(1.05, 3.64)	
**T**CT			36	796	7.8	15.3	5.15	< 0.001
			(Thr, Ser)	(Gly, Gly)			(2.53, 10.48)	
**C**CG → **T**CG	nt1485	B cell epitope	38	798	16.8	29.9	2.40	0.001
			(Pro, Ser)	(Tyr, Tyr)			(1.41, 4.08)	
CC**G** → CC**A**	nt1511							0.121
**G**CC → **A**CC	nt1569							0.511
C**G**C → C**A**C	nt1630							0.463
CG/A**C** → CG/A**T**	nt1631	CP, NRE	86		1.8	8.3	4.09	0.01
			(His, His)				(1.41, 11.85)	
**C**AT → **T**AT	nt1653	Box α, CP,	94		18.6	35.4	2.58	< 0.001
		C/EBP, Enh2	(His, Tyr)				(1.59, 4.19)	
**A**CC → **T**CC	nt1689							
**G**TG → **T**TG	nt1719	BH3-like motif,	116		57.6	82.6	2.11	0.01
		CP, Enh2, HNF3,	(Val, Leu)				(1.19, 3.73)	
		T cell epitope						
TT**A** → TT**G**	nt1721							0.696
AG**A** → AG**G**	nt1757							0.304
TT**G** → TT**A**	nt1775							0.353
**T**GC → **C**GC	nt1800	CP	143		0.3	3.5	23.59	0.008
			(Cys, Arg)				(2.25, 247.65)	

^a^Mutated nucleotides are shown in bold.

B cell epitope: region (aa positions 29–48); BH3-like motif: region (aa positions 116–132); Box α, region (nt1646-1668); C/EBP, CCAAT/enhancing binding protein, region (nt1643-1658); CP, core promoter, region (nt1613-1849); Enh2: enhancer 2, region (nt1636-1744); HNF3, hepatocyte nuclear factor 3, region (nt1713-1723); NRE, negative regulatory element, region (nt1611-1634); T-cell epitope: region (aa positions 116–127).

Here, how those seven genotype C HBx risk mutations, A1383C, R1479C/T, C1485T, C1631T, C1653T, G1719T, and T1800C, are distributed among genotype C HCC patients from different countries is an intriguing question. We therefore extracted HCC sequences from Japan, Mainland China, South Korea and Philippines, which constitute the majority of our enrolled genotype C HCC sequences (143/144, 99%) in S4 Table. Interestingly, three of these mutations had different distributions (1383C, *P* < 0.001; 1479Y, *P* < 0.001; 1653T, *P* = 0.003) among four countries. 1383C was predominant among HCC patients from South Korea (18/23, 78.3%) and Philippines (2/2, 100%). Around half of HCC patients from Japan and South Korea possessed 1479C (51.9% and 56.5%, respectively) but only those from Japan carried the 1479T mutation (22/106, 20.8%). 1653T seemed to be more prevalent among HCC patients from Japan (46/106, 43.4%) and Philippines (1/2, 50%). Only 8.3% (1/12) of HCC patients from Mainland China possessed 1653T mutation.

### Distribution of genotype C HBV-related HCC risk mutations among different disease phases

**Fig 2:**
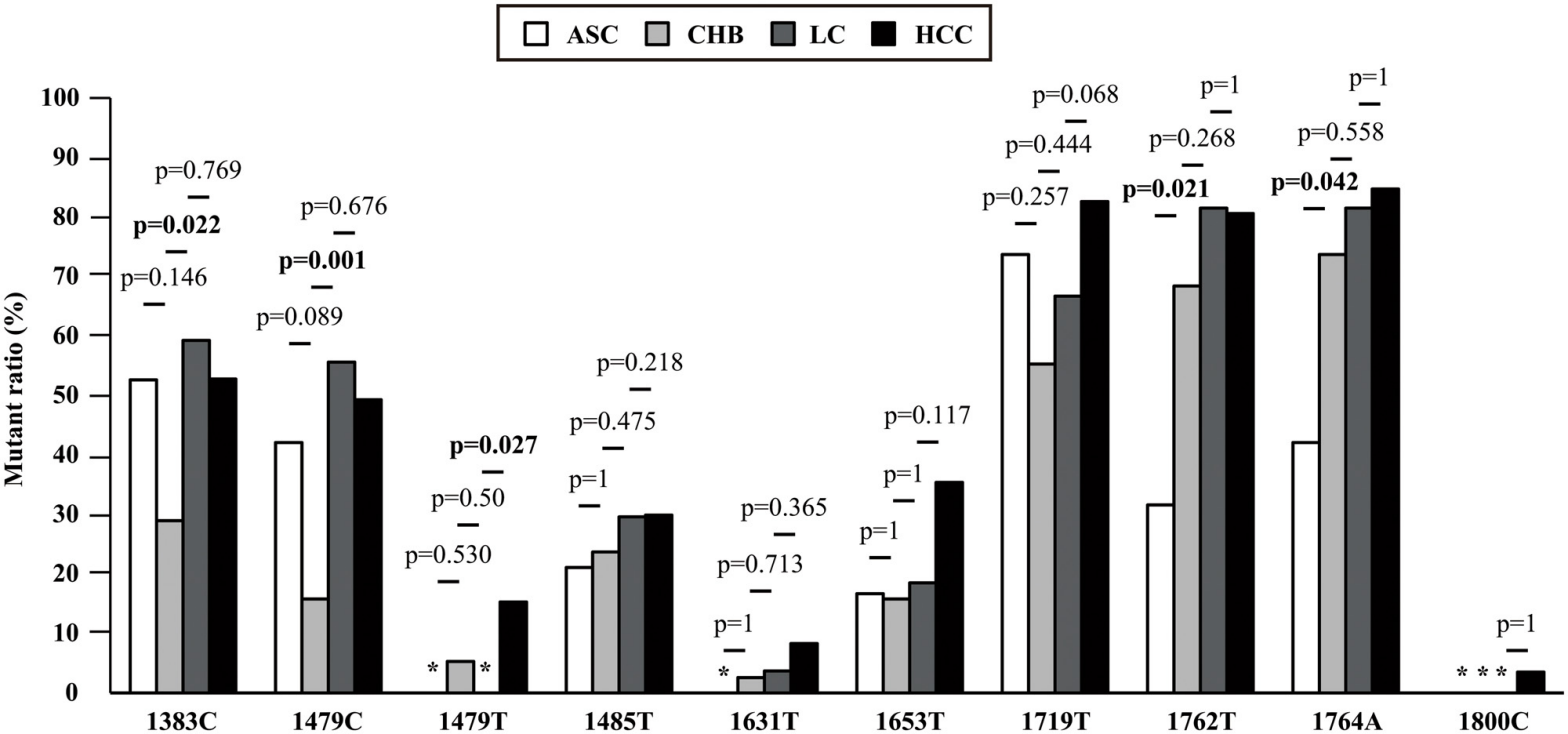
HCC risk mutations in genotype C sequences across different disease phases. We checked the distribution of HBV genotype C HCC risk mutations among sequences from different disease phases (ASC, 18; CHB, 38; LC, 27; HCC, 144). Those mutations showed characteristics among different phases: 1) except three mutations (1479T, 1631T, and 1800C) all the other mutations pre-existed in ASC, among which 1383C and 1719T were most pronounced. More than 50% ASC possessed either one of these two mutations and 42% ASC possessed both; 2) the frequency of four mutations (1485T, 1631T, 1762T, and 1764A) showed an increasing trend accompanied with the disease progression though the changes among groups were not significant (Jonckheere-Terpstra trend test, *P* > 0.05); and 3) ratios of 4 mutations (1383C, 1479C, 1479T, and 1719T) fluctuated among different disease phases. No mutants were observed in the cases denoted by asterisks.

In order to know the trend of those mutations in the progression to HCC, we checked their distribution in genotype C sequences from different disease phases (ASC, 18; CHB, 38; LC, 27; and HCC, 144) (Fig 2). Since the double basal core promoter mutations (BCP) 1762T/1764A were reported to be associated with HCC in previous reports frequently [11,12] though they were with p values 0.079 and 0.190 and not selected as significant ones in Table 3, we also included these two positions into the analyses as reference here. We found several interesting characters: 1) except three mutations (1479T, 1631T, and 1800C) all the other mutations pre-existed in ASC, among which 1383C and 1719T were most pronounced. More than 50% ASC possessed either one of these two mutations and 42% ASC possessed both; 2) the frequency of four mutations (1485T, 1631T, 1762T, and 1764A) showed an increasing trend accompanied with the disease progression though the changes among groups were not significant (Jonckheere-Terpstra trend test, *P* > 0.05); and 3) ratios of 4 mutations (1383C, 1479C, 1479T, and 1719T) fluctuated among different disease phases.

### Distribution of genotype C HCC risk nucleotides among different genotypes

**Fig 3:**
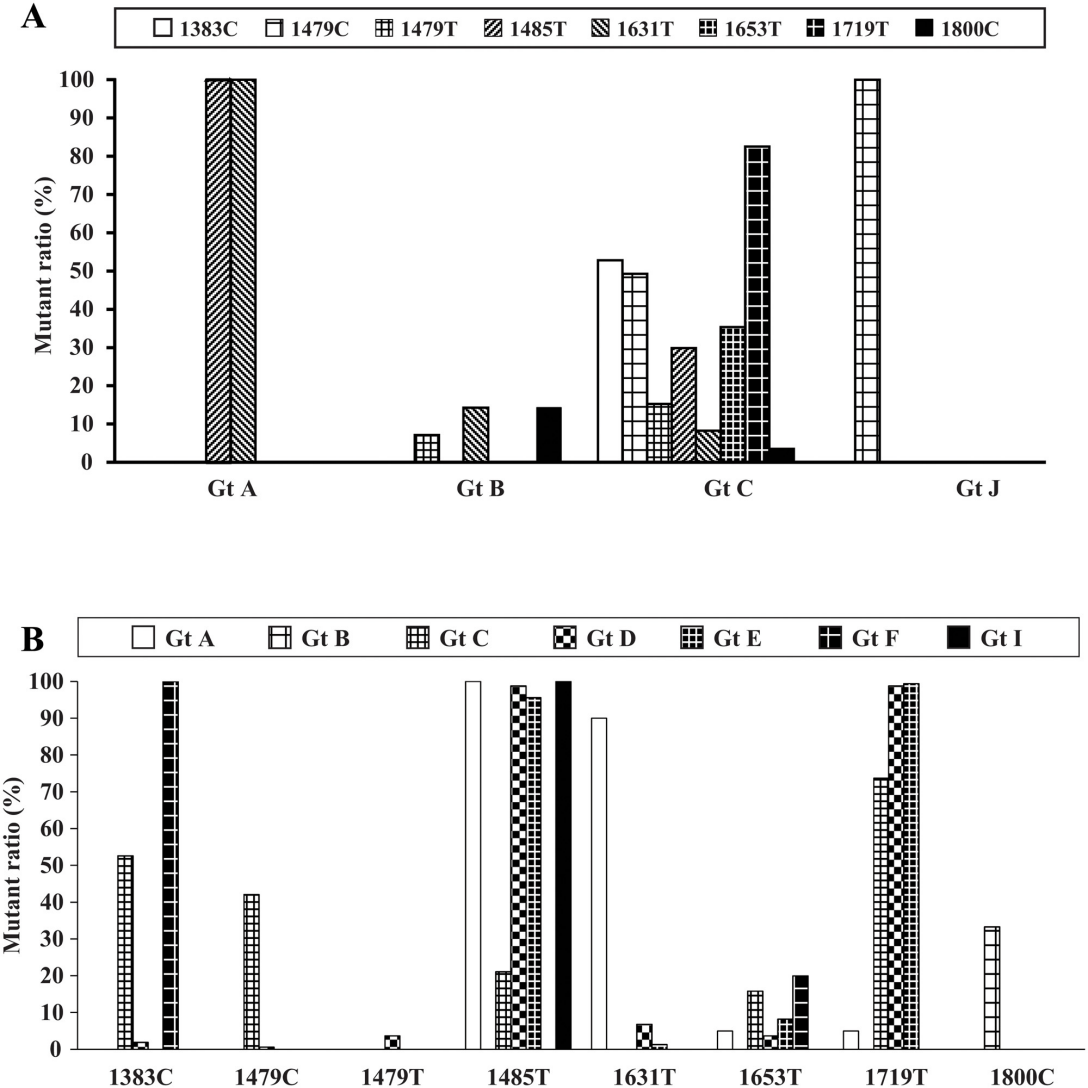
Distribution of genotype C HBx mutations for HCC. The data among HCC with different genotypes (A) and among ASC with different genotypes (B) were analyzed. (A) In genotype A HCC sequences (n = 2), two positions (1485T and 1631T) showed 100% genotype C HCC risk nucleotides. Genotype J HCC sequence (n = 1) presented one position with 100% genotype C HCC risk nucleotide (1479C). In genotype B HCC sequences (n = 14), three positions (1479T, 1631T and 1800C) showed the genotype C HCC risk nucleotides at relatively lower ratios less than 20%. (B) In ASC sequences (genotype A: 20, genotype B: 3, genotype D: 161, genotype E: 158, genotype F: 5, genotype I: 1), 1485T were frequently observed in genotypes A, D, E, and I, and 1631T and 1800C were in genotype A (90%) and B (33.3%), respectively. 1383C and 1719T were seen in genotype D and F, respectively, together with genotype C.

In order to know if genotype C HCC risk mutations were common events also in other genotypes, we checked the distribution of those mutations in our enrolled sequences with other genotypes. In Fig 3A we investigated other genotypes in HCC, and risk mutations found in genotype C at two positions 1485T and 1631T were 100% in genotype A HCC sequences (n = 2) and 1479C was 100% in genotype J HCC sequence (n = 1). In genotype B HCC sequences (n = 14), three positions (1479T, 1631T and 1800C) showed the genotype C HCC risk nucleotides while they were all at relatively lower ratios less than 20%. Through the analyses on the AA substitutions found in genotype C HCC (S3 Table), the appearance of the nonsynonymous mutations above in genotype A and J were confirmed in HCC (S5 Table). Consistently the data indicated that risk mutations identified in Genotype C were also associated with HCC across genotypes.

Meanwhile it was also interesting to ask whether the risk mutations were related to carcinogenic potentials of individual genotypes. To examine this, we analyzed other genotypes in ASC (genotype A: 20, genotype B: 3, genotype D: 161, genotype E: 158, genotype F: 5, genotype I: 1) (Fig 3B). While 1485T was observed in almost all the genotypes, 1631T and 1800C were seen in 90% and 33.3% of genotype A and B, respectively. Remarkably, genotype D presented 1719T (98.8%) and genotype F presented 1383C (100%) together with genotype C, and in consideration for the highly carcinogenic properties of genotypes D and F as well as C speculating those mutations’ participation in HCC development would be of interest [4]. Again through the analyses on the AA substitutions found in genotype C HCC (S3 Table), the emergence of nonsynonymous mutations in the genotypes examined above were supported (S6 Table). Overall risk mutations screened in our analyses on genotype C were indicated to be associated with hepatocarcinogenesis in multiple genotypes as well.

## Discussion

HBV X region contains only 465 bp (nt1374-1838) and encodes the 16.5 kDa HBx protein, which partially overlaps with the RNase H part of HBV Polymerase at C terminus, and also contains several critical cis-elements. The three-dimensional structure of HBx is still unknown, while there have been data showing that this unstructured protein could gain secondary structure under certain conditions and therefore play roles via the interaction with target proteins [13]. Therefore, genetic alterations in this region may not only affect the reading frame of HBx but also the overlapped cis-elements and the possible binding affinities of this protein to targets.

In this study, we compared HBx sequences between genotype C-infected HCC and Non-HCC patients. Sixteen nucleotide differences between the two groups were found and seven of them (A1383C, R1479Y, C1485T, C1631T, C1653T, G1719T, and T1800C) were further identified to be critical for genotype C HBV-related HCC, also observed in AA analyses in parallel as for the nonsynonymous mutations.

Nt1383 was located in the negative regulation domain of HBx (aa 1–50), and this 1383C mutation was first found to be associated with HCC in a Korean cohort and later was found to be able to induce a higher NF-kB activity in transformed cells [14,15]. In one Chinese clinical study, 1383C was also associated with worse prognosis of patients after liver transplantation [16]. Very recently, a group reported that microRNA 15a/16 (miR-15a/16), a tumor suppressor, could directly target wild HBx RNA sequence (nt1362-1383), thus consequently induced Bcl-2 expression. The miR-15a/16 binding ability declined significantly when mutations were introduced into the wild HBx, including one at nt1383 [17]. Thus, on the one hand this mutation could prevent the infected cell from apoptosis by altering critical cell signal pathways, and on the other hand it may also regulate viral replication since the location was within miRNA binding sites and therefore interfere with the competitive binding activity among host mRNAs, miRNAs, and viral RNAs [18].

Both nt1479 and nt1485 are located in B cell epitope region of HBx while the mutant frequency of nt1479 was much higher than nt1485, which indicated that nt1479 may face higher immune pressure than did nt1485, though the mechanism still needs further clarification. 1653T mutation was firstly identified among Anti-HBe positive carriers who underwent acute exacerbation in Japan [19]. One year later this mutation was also found in HCC patients [20]. One group revealed that this mutation, together with 1762T/1764A mutations, could reduce the level of pre-C mRNA to around 55% and e-antigen secretion by a transient transfection system using Huh7 cells [21]. However, the association between this 1653T mutation and carcinogenesis was still unclear. One possible mechanism is those mutations located in the transactivation domain of HBx (aa 51–140) such as 1631T, 1653T and 1719T playing regulatory roles in carcinogenesis via multiple cis-elements [22]. For instance, nt1719 located in a BH3-like motif (aa116-132) of HBx sequence, through which HBx bound to CED-9, a homolog of Bcl-2 with the effects of pro-apoptosis [23]. *In vivo* and *in vitro* experiments had shown that the binding affinity of HBx to CED-9 could be abolished by alternating residues in the BH-3 like motif of HBx [23,24]. Interestingly, by the same BH-3 like motif, HBx could interact with two other Bcl-2 family members, Bcl-2 and Bcl-xL, and such interactions are critical for HBx to increase intracellular calcium concentration, which is required for viral replication and cell death [25]. Thus, it is possible that mutations emerging in this BH3-like motif may be carcinogenetic potentially affecting those interactions above.

The mutation occurring in nt1800 is a novel HCC risk mutation identified in our study. So far the function of this mutation in carcinogenesis remains unclear. However, a recent genome-wide analysis which investigated HBV integration sites in 88 Chinese HCC patients had drawn our attention. The author reported that almost 40% of the integrated HBV genomes were cleaved around at nt1800 [26]. Such a high frequency of breakage near one position indicated a potential role of this site in carcinogenesis since HBV genome integration had long been considered as an important factor in the development of HCC. Thus, mutation on this position possibly affects the integration though we are still not certain about its role. Moreover, HBV genome integration is currently believed to be an early event in HBV chronic infection. Thus, patients’ sequences which possessed this mutation in ASC phase possibly had a higher risk of HCC. From our data, we found that only genotype B ASC possessed a certain frequency of 1800C whereas other genotypes (A, C, D, E, F, and I) did not. In addition, several clinical studies also revealed that genotype B tended to develop more HCC in younger patients than did genotype C but the mechanism was not yet known [9,27]. It is possible that this position suspected of integration may accelerate the carcinogenesis in genotype B HBV-infected patients.

Previously, in especially genotype C among several genotypes, the well-known double BCP mutations were reported to be universal ones for risk of advanced liver diseases including HCC [28–31]. While in our enrolled sequences, we could not find significant difference of BCP mutations between genotype C HCC and Non-HCC (1762T, *P* = 0.079; 1764A, *P* = 0.190). One possible explanation maybe the pre-existence of BCP mutations in earlier disease phases, that is, that virus may have mutated soon after the infection or patients were initially infected with mutant type. We then checked BCP mutation ratio in different disease phases. As shown in Fig 2, BCP mutant ratio increased obviously from ASC to CHB (1762T, ASC 33.3%, CHB 68.4%, *P* = 0.02; and 1764A, ASC 44.4%, CHB 73.7%, *P* = 0.04), while it seemed to reach the plateau from CHB phase. The differences between CHB and LC, and LC and HCC were not significant though the mutant ratio kept elevating accompanied with disease progression. We speculated that the two mutations might occur in the earlier stage of disease and act as a “driver mutation” or “first-hit” throughout the long period of carcinogenesis.

Similar trends were also found in other nucleotide positions. For instance, the ratio of 1485T among different disease phases (ASC, CHB, LC and HCC) was 21.1%, 23.7%, 29.6% and 29.9%, respectively. 1631T possessed 0% in ASC, 2.6% in CHB, 3.7% in LC and 8.3% in HCC. In addition, several other positions (4/465, data not shown) also presented similar trends though those positions have not been verified to increase HCC risk. Different from this format, the ratio of 1383C and 1479C/T fluctuated among disease phases. For instance, the mutant ratio of 1383C in genotype C ASC and HCC sequences were similar (ASC: 52.6%, HCC: 52.8%) while this site showed drastic changes from ASC to CHB, and CHB to LC (CHB: 28.9%, LC: 59.5%). 158 out of the whole 465 positions also showed the similar format. In addition, the remaining 301 nucleotide positions of HBx kept constant.

Many mutations occurring in HBV sequence during the treatment disappeared after withdrawal of antiviral treatment [32]. One group also reported that some nucleotide positions seemed to play inverse roles during the processions from CHB to LC and from LC to HCC [33]. In our study, by comparing HBx sequences among different disease groups, we found that the changes of HBx sequences could also be considered as a “multi-step and multi-factor” procession. During chronic infection, some positions kept conserved nucleotides or kept developing mutant nucleotides, i.e., nt1762, 1764, etc. We consider those positions may possess a constantly favored nucleotide type *in vivo*, but some positions changing their nucleotides across disease phases may possess a transiently favored type, as it were. For instance, HBV may produce new functional binding sites for molecules such as transcription factors and miRNAs or frame shifts by temporarily mutating some positions. The existing period of such type of mutation depends on the balance among host factors, microenvironments and viruses themselves.

Several genotype C HCC risk mutations were pre-existing in other genotypes. For instance, 1485T seemed to be a very frequent event in ASC with many genotypes (genotype A, D, E and I). Genotype A possessed a ratio of 1485T for more than 90% either in ASC or HCC, but the ratios within genotype B ASC and HCC were zero. In addition, ASC with genotypes D and E showed high ratios for 1719T though we had no related HCC data. Besides, genotype B ASC got the highest 1800C ratio among all the genotypes. Whether these mutations in other genotypes were associated with the risk for HCC remains to be investigated. Such distinct differences among genotypes may serve to develop disease predicting tools in near future.

Taken together, nucleotides 1383C, 1479Y, 1485T, 1631T, 1653T, 1719T, and 1800C in HBV X region were independent risk factors for HBV genotype C-infected HCC patients. But some of them also pre-existed in other HBV genotypes, even as major types. Mutations associated with HCC risk were mainly located in HBx transactivation domain, viral promoter, protein/miRNA binding sites, and the area for immune epitopes. Moreover, the signatures of these mutations were unique to disease phases leading to HCC, suggesting molecular counteractions between the virus and host during hepatocarcinogenesis. Although a large number of enrolled sequences could to some extent weaken the bias, there still inevitably exist many limitations in our study such as the population heterogeneity, selection bias, small number of cases with genotypes other than genotype C deposited in the database and a lot of missing data on demographic information. Therefore, further longitudinal studies are needed to verify the roles of these mutations in earlier disease stages and the process of oncogenesis with the interactive effects of other host factors such as age and gender and viral factors such as HBeAg status. Our study would shed light on early diagnoses and interventions for genotype C HBV-infected patients suffering from the high-risk of HCC.

## Supporting Information

S1 Table One thousand one hundred fifteen HBx sequences with genotype and diagnosis information. (DOC)

S2 Table Fifty seven references with PUBMED ID or DOI number. (DOC)

S3 Table Amino acid differences between HCC and Non-HCC patients with HBV genotype C infection. (DOC)

S4 Table Distribution of genotype C HCC risk mutations among four Asian countries. (DOC)

S5 Table Distribution of genotype C HCC risk amino acid residues across different HBV genotypes in HCC patients. (DOC)

S6 Table Distribution of genotype C HCC risk amino acid residues across AsC with different HBV genotypes. (DOC)
