# Supplementary material for: The Characteristic Changes in Hepatitis B Virus X Region for Hepatocellular Carcinoma: A Comprehensive Analysis Based on Global Data
Source: PLoS One. 2015 May 5;10(5):e0125555. doi: 10.1371/journal.pone.0125555 (PMC4420286; doi:10.1371/journal.pone.0125555)
Supplement: S1 Table — (DOC) [file pone.0125555.s001.doc]

**S1 Table. One thousand one hundred fifteen HBx sequences with genotype and diagnosis information.**

| Accession number | Genotypes | With HCC diagnosis (0, NO; 1, YES) |
| --- | --- | --- |
| AB194950 | A | 0 |
| AB194951 | A | 0 |
| AB194952 | A | 0 |
| AB222707 | A | 0 |
| AY233275 | A | 0 |
| AY233276 | A | 0 |
| AY233277 | A | 0 |
| AY233278 | A | 0 |
| AY233280 | A | 0 |
| AY233281 | A | 0 |
| AY233282 | A | 0 |
| AY233285 | A | 0 |
| AY233286 | A | 0 |
| AY233288 | A | 0 |
| AY233289 | A | 0 |
| AY233290 | A | 0 |
| EU410082 | A | 0 |
| GQ161813 | A | 0 |
| AB241115 | A | 0 |
| AY373428 | A | 0 |
| AY373429 | A | 0 |
| AY373432 | A | 0 |
| AY161140 | A | 0 |
| AY161147 | A | 0 |
| HM011485 | A | 0 |
| FJ349222 | A | 0 |
| AJ344115 | A | 0 |
| AY161138 | A | 0 |
| AY161142 | A | 0 |
| AY161145 | A | 0 |
| JQ687529 | A | 0 |
| JQ687533 | A | 0 |
| AB014370 | A | 1 |
| AB241114 | A | 1 |
| FJ904411 | A | 0 |
| FJ904434 | A | 0 |
| AB365445 | B | 0 |
| DQ995803 | B | 0 |
| DQ995804 | B | 0 |
| AB195933 | B | 0 |
| JN406371 | B | 0 |
| FJ349236 | B | 0 |
| AY167089 | B | 0 |
| HM011487 | B | 0 |
| HM011492 | B | 0 |
| HM011494 | B | 0 |
| HM011496 | B | 0 |
| HM011498 | B | 0 |
| HM011503 | B | 0 |
| HM011504 | B | 0 |
| JQ429079 | B | 0 |
| HM011466 | B | 0 |
| HM011470 | B | 0 |
| HM011471 | B | 0 |
| HM011473 | B | 0 |
| HM011475 | B | 0 |
| HM011476 | B | 0 |
| HM011477 | B | 0 |
| HM011478 | B | 0 |
| HM011483 | B | 0 |
| FJ386582 | B | 0 |
| FJ386583 | B | 0 |
| FJ386584 | B | 0 |
| FJ386600 | B | 0 |
| FJ386608 | B | 0 |
| FJ386610 | B | 0 |
| FJ386615 | B | 0 |
| FJ386634 | B | 0 |
| FJ386636 | B | 0 |
| FJ386642 | B | 0 |
| FJ386648 | B | 0 |
| FJ386654 | B | 0 |
| FJ386655 | B | 0 |
| FJ386656 | B | 0 |
| FJ386658 | B | 0 |
| FJ386660 | B | 0 |
| FJ386666 | B | 0 |
| FJ386668 | B | 0 |
| FJ386669 | B | 0 |
| FJ386675 | B | 0 |
| FJ386676 | B | 0 |
| FJ386680 | B | 0 |
| FJ386681 | B | 0 |
| FJ386682 | B | 0 |
| FJ386683 | B | 0 |
| FJ386684 | B | 0 |
| FJ386688 | B | 0 |
| FJ562219 | B | 0 |
| FJ562222 | B | 0 |
| FJ562224 | B | 0 |
| FJ562231 | B | 0 |
| FJ562234 | B | 0 |
| FJ562236 | B | 0 |
| FJ562237 | B | 0 |
| FJ562240 | B | 0 |
| FJ562246 | B | 0 |
| FJ562253 | B | 0 |
| FJ562254 | B | 0 |
| FJ562257 | B | 0 |
| FJ562259 | B | 0 |
| FJ562260 | B | 0 |
| FJ562262 | B | 0 |
| FJ562289 | B | 0 |
| FJ562296 | B | 0 |
| FJ562303 | B | 0 |
| FJ562311 | B | 0 |
| FJ562312 | B | 0 |
| FJ562316 | B | 0 |
| FJ562321 | B | 0 |
| FJ562322 | B | 0 |
| AY167097 | B | 0 |
| AY167098 | B | 0 |
| AB241116 | B | 1 |
| AB241117 | B | 1 |
| JQ429080 | B | 1 |
| JQ429081 | B | 1 |
| DQ995801 | B | 1 |
| DQ995802 | B | 1 |
| AY163870 | B | 1 |
| AY206373 | B | 1 |
| AY206375 | B | 1 |
| AY206380 | B | 1 |
| AY206383 | B | 1 |
| AY206387 | B | 1 |
| AY206390 | B | 1 |
| AY206391 | B | 1 |
| AB033556 | C | 0 |
| AB222714 | C | 0 |
| AB365451 | C | 0 |
| AB365452 | C | 0 |
| AB485808 | C | 0 |
| S75184 | C | 0 |
| X04615 | C | 0 |
| AY641558 | C | 0 |
| AY641560 | C | 0 |
| AY641561 | C | 0 |
| EU410079 | C | 0 |
| EU410080 | C | 0 |
| EU410081 | C | 0 |
| EU670263 | C | 0 |
| AJ748098 | C | 0 |
| AY057947 | C | 0 |
| D28880 | C | 0 |
| AB195930 | C | 0 |
| AB195936 | C | 0 |
| AB195939 | C | 0 |
| AB195942 | C | 0 |
| AB195945 | C | 0 |
| AB195947 | C | 0 |
| AB195949 | C | 0 |
| AB195952 | C | 0 |
| AB195955 | C | 0 |
| AB241109 | C | 0 |
| AB250109 | C | 0 |
| GQ475305 | C | 0 |
| GQ475306 | C | 0 |
| GQ475307 | C | 0 |
| GQ475308 | C | 0 |
| GQ475309 | C | 0 |
| GQ475310 | C | 0 |
| GQ475311 | C | 0 |
| GQ475312 | C | 0 |
| GQ475313 | C | 0 |
| GQ475314 | C | 0 |
| GQ475315 | C | 0 |
| GQ475316 | C | 0 |
| GQ475317 | C | 0 |
| GQ475318 | C | 0 |
| GQ475319 | C | 0 |
| GQ475320 | C | 0 |
| GQ475321 | C | 0 |
| JQ429078 | C | 0 |
| FJ349225 | C | 0 |
| AY247030 | C | 0 |
| AY247031 | C | 0 |
| AY247032 | C | 0 |
| HQ622095 | C | 0 |
| DQ536410 | C | 0 |
| AY167090 | C | 0 |
| AY167092 | C | 0 |
| EF384201 | C | 0 |
| AB241110 | C | 0 |
| AB241111 | C | 0 |
| GQ475322 | C | 0 |
| GQ475324 | C | 0 |
| GQ475325 | C | 0 |
| GQ475326 | C | 0 |
| GQ475327 | C | 0 |
| GQ475328 | C | 0 |
| GQ475329 | C | 0 |
| GQ475330 | C | 0 |
| GQ475331 | C | 0 |
| GQ475332 | C | 0 |
| GQ475333 | C | 0 |
| GQ475334 | C | 0 |
| GQ475335 | C | 0 |
| GQ475336 | C | 0 |
| GQ475337 | C | 0 |
| GQ475338 | C | 0 |
| HM011486 | C | 0 |
| HM011488 | C | 0 |
| HM011489 | C | 0 |
| HM011491 | C | 0 |
| HM011493 | C | 0 |
| HM011497 | C | 0 |
| HM011500 | C | 0 |
| HM011501 | C | 0 |
| HM011502 | C | 0 |
| AB048705 | C | 0 |
| DQ089756 | C | 0 |
| DQ089757 | C | 0 |
| DQ089758 | C | 0 |
| DQ089759 | C | 0 |
| DQ089760 | C | 0 |
| DQ089761 | C | 0 |
| DQ089762 | C | 0 |
| DQ089763 | C | 0 |
| DQ089764 | C | 0 |
| DQ089765 | C | 0 |
| DQ089766 | C | 0 |
| DQ089767 | C | 0 |
| DQ089768 | C | 0 |
| DQ089769 | C | 0 |
| DQ089770 | C | 0 |
| DQ089771 | C | 0 |
| DQ089772 | C | 0 |
| DQ089773 | C | 0 |
| DQ089775 | C | 0 |
| DQ089776 | C | 0 |
| DQ089778 | C | 0 |
| DQ089780 | C | 0 |
| DQ089781 | C | 0 |
| DQ089782 | C | 0 |
| DQ089783 | C | 0 |
| DQ089784 | C | 0 |
| DQ089785 | C | 0 |
| DQ089787 | C | 0 |
| DQ089788 | C | 0 |
| DQ089789 | C | 0 |
| DQ089790 | C | 0 |
| DQ089791 | C | 0 |
| DQ089792 | C | 0 |
| DQ089793 | C | 0 |
| DQ089794 | C | 0 |
| DQ089795 | C | 0 |
| DQ089796 | C | 0 |
| DQ089797 | C | 0 |
| DQ089798 | C | 0 |
| DQ089799 | C | 0 |
| DQ089800 | C | 0 |
| DQ089801 | C | 0 |
| DQ089802 | C | 0 |
| DQ089803 | C | 0 |
| DQ089804 | C | 0 |
| HM011465 | C | 0 |
| HM011468 | C | 0 |
| HM011479 | C | 0 |
| HM011481 | C | 0 |
| JN418493 | C | 0 |
| JN418494 | C | 0 |
| JN418495 | C | 0 |
| JN418496 | C | 0 |
| JN418497 | C | 0 |
| JN418498 | C | 0 |
| JN418499 | C | 0 |
| JN418500 | C | 0 |
| JN418501 | C | 0 |
| JN418502 | C | 0 |
| JN418503 | C | 0 |
| JN418504 | C | 0 |
| JN418505 | C | 0 |
| JN418506 | C | 0 |
| JN418507 | C | 0 |
| JN418508 | C | 0 |
| JN418509 | C | 0 |
| JN418510 | C | 0 |
| JN418511 | C | 0 |
| JN418512 | C | 0 |
| JN418513 | C | 0 |
| JN418514 | C | 0 |
| JN418515 | C | 0 |
| JN418516 | C | 0 |
| JN418517 | C | 0 |
| JN418518 | C | 0 |
| JN418519 | C | 0 |
| JN418520 | C | 0 |
| JN418521 | C | 0 |
| JN418522 | C | 0 |
| JN418523 | C | 0 |
| JN418524 | C | 0 |
| JN418525 | C | 0 |
| JN418526 | C | 0 |
| JN418527 | C | 0 |
| JN418528 | C | 0 |
| FJ386574 | C | 0 |
| FJ386575 | C | 0 |
| FJ386576 | C | 0 |
| FJ386577 | C | 0 |
| FJ386578 | C | 0 |
| FJ386579 | C | 0 |
| FJ386580 | C | 0 |
| FJ386581 | C | 0 |
| FJ386585 | C | 0 |
| FJ386586 | C | 0 |
| FJ386587 | C | 0 |
| FJ386588 | C | 0 |
| FJ386589 | C | 0 |
| FJ386591 | C | 0 |
| FJ386592 | C | 0 |
| FJ386593 | C | 0 |
| FJ386594 | C | 0 |
| FJ386595 | C | 0 |
| FJ386596 | C | 0 |
| FJ386597 | C | 0 |
| FJ386598 | C | 0 |
| FJ386599 | C | 0 |
| FJ386601 | C | 0 |
| FJ386602 | C | 0 |
| FJ386603 | C | 0 |
| FJ386604 | C | 0 |
| FJ386605 | C | 0 |
| FJ386606 | C | 0 |
| FJ386607 | C | 0 |
| FJ386609 | C | 0 |
| FJ386611 | C | 0 |
| FJ386612 | C | 0 |
| FJ386613 | C | 0 |
| FJ386614 | C | 0 |
| FJ386616 | C | 0 |
| FJ386617 | C | 0 |
| FJ386618 | C | 0 |
| FJ386619 | C | 0 |
| FJ386620 | C | 0 |
| FJ386621 | C | 0 |
| FJ386622 | C | 0 |
| FJ386623 | C | 0 |
| FJ386624 | C | 0 |
| FJ386625 | C | 0 |
| FJ386626 | C | 0 |
| FJ386627 | C | 0 |
| FJ386628 | C | 0 |
| FJ386629 | C | 0 |
| FJ386630 | C | 0 |
| FJ386631 | C | 0 |
| FJ386632 | C | 0 |
| FJ386633 | C | 0 |
| FJ386635 | C | 0 |
| FJ386637 | C | 0 |
| FJ386638 | C | 0 |
| FJ386639 | C | 0 |
| FJ386640 | C | 0 |
| FJ386641 | C | 0 |
| FJ386643 | C | 0 |
| FJ386644 | C | 0 |
| FJ386645 | C | 0 |
| FJ386646 | C | 0 |
| FJ386647 | C | 0 |
| FJ386649 | C | 0 |
| FJ386650 | C | 0 |
| FJ386651 | C | 0 |
| FJ386652 | C | 0 |
| FJ386653 | C | 0 |
| FJ386657 | C | 0 |
| FJ386659 | C | 0 |
| FJ386661 | C | 0 |
| FJ386662 | C | 0 |
| FJ386663 | C | 0 |
| FJ386664 | C | 0 |
| FJ386665 | C | 0 |
| FJ386667 | C | 0 |
| FJ386670 | C | 0 |
| FJ386671 | C | 0 |
| FJ386672 | C | 0 |
| FJ386673 | C | 0 |
| FJ386674 | C | 0 |
| FJ386677 | C | 0 |
| FJ386678 | C | 0 |
| FJ386679 | C | 0 |
| FJ386685 | C | 0 |
| FJ386686 | C | 0 |
| FJ386687 | C | 0 |
| FJ386689 | C | 0 |
| FJ562218 | C | 0 |
| FJ562220 | C | 0 |
| FJ562221 | C | 0 |
| FJ562223 | C | 0 |
| FJ562225 | C | 0 |
| FJ562226 | C | 0 |
| FJ562227 | C | 0 |
| FJ562228 | C | 0 |
| FJ562229 | C | 0 |
| FJ562232 | C | 0 |
| FJ562233 | C | 0 |
| FJ562235 | C | 0 |
| FJ562238 | C | 0 |
| FJ562239 | C | 0 |
| FJ562241 | C | 0 |
| FJ562242 | C | 0 |
| FJ562243 | C | 0 |
| FJ562244 | C | 0 |
| FJ562245 | C | 0 |
| FJ562247 | C | 0 |
| FJ562248 | C | 0 |
| FJ562249 | C | 0 |
| FJ562250 | C | 0 |
| FJ562251 | C | 0 |
| FJ562252 | C | 0 |
| FJ562255 | C | 0 |
| FJ562256 | C | 0 |
| FJ562258 | C | 0 |
| FJ562261 | C | 0 |
| FJ562264 | C | 0 |
| FJ562265 | C | 0 |
| FJ562266 | C | 0 |
| FJ562267 | C | 0 |
| FJ562268 | C | 0 |
| FJ562269 | C | 0 |
| FJ562270 | C | 0 |
| FJ562271 | C | 0 |
| FJ562272 | C | 0 |
| FJ562273 | C | 0 |
| FJ562274 | C | 0 |
| FJ562275 | C | 0 |
| FJ562276 | C | 0 |
| FJ562277 | C | 0 |
| FJ562278 | C | 0 |
| FJ562279 | C | 0 |
| FJ562280 | C | 0 |
| FJ562281 | C | 0 |
| FJ562282 | C | 0 |
| FJ562283 | C | 0 |
| FJ562284 | C | 0 |
| FJ562285 | C | 0 |
| FJ562287 | C | 0 |
| FJ562288 | C | 0 |
| FJ562290 | C | 0 |
| FJ562291 | C | 0 |
| FJ562292 | C | 0 |
| FJ562293 | C | 0 |
| FJ562294 | C | 0 |
| FJ562295 | C | 0 |
| FJ562297 | C | 0 |
| FJ562298 | C | 0 |
| FJ562299 | C | 0 |
| FJ562300 | C | 0 |
| FJ562301 | C | 0 |
| FJ562302 | C | 0 |
| FJ562304 | C | 0 |
| FJ562305 | C | 0 |
| FJ562306 | C | 0 |
| FJ562307 | C | 0 |
| FJ562308 | C | 0 |
| FJ562310 | C | 0 |
| FJ562313 | C | 0 |
| FJ562314 | C | 0 |
| FJ562315 | C | 0 |
| FJ562317 | C | 0 |
| FJ562318 | C | 0 |
| FJ562319 | C | 0 |
| FJ562320 | C | 0 |
| FJ562323 | C | 0 |
| FJ562324 | C | 0 |
| FJ562325 | C | 0 |
| FJ562326 | C | 0 |
| FJ562327 | C | 0 |
| FJ562328 | C | 0 |
| FJ562329 | C | 0 |
| FJ562330 | C | 0 |
| FJ562331 | C | 0 |
| FJ562332 | C | 0 |
| FJ562333 | C | 0 |
| FJ562334 | C | 0 |
| FJ562335 | C | 0 |
| FJ562336 | C | 0 |
| FJ562337 | C | 0 |
| FJ562339 | C | 0 |
| FJ562340 | C | 0 |
| AB670274 | C | 0 |
| AB670276 | C | 0 |
| AB670277 | C | 0 |
| AB670278 | C | 0 |
| AB670279 | C | 0 |
| AB670280 | C | 0 |
| AB670281 | C | 0 |
| AB670282 | C | 0 |
| AB670283 | C | 0 |
| AB670284 | C | 0 |
| AB670287 | C | 0 |
| AB670288 | C | 0 |
| AB670289 | C | 0 |
| AB670290 | C | 0 |
| AB670291 | C | 0 |
| AB670292 | C | 0 |
| AB670294 | C | 0 |
| AB670295 | C | 0 |
| AB670296 | C | 0 |
| AB670297 | C | 0 |
| AB670298 | C | 0 |
| AB670300 | C | 0 |
| AB670301 | C | 0 |
| AB670302 | C | 0 |
| AB670303 | C | 0 |
| AB670304 | C | 0 |
| AB670305 | C | 0 |
| AB670306 | C | 0 |
| AB670307 | C | 0 |
| AB670308 | C | 0 |
| AB670309 | C | 0 |
| AB670310 | C | 0 |
| AB670311 | C | 0 |
| AY167096 | C | 0 |
| AY167099 | C | 0 |
| AB014360 | C | 1 |
| AB014361 | C | 1 |
| AB014362 | C | 1 |
| AB014363 | C | 1 |
| AB014364 | C | 1 |
| AB014365 | C | 1 |
| AB014367 | C | 1 |
| AB014368 | C | 1 |
| AB014369 | C | 1 |
| AB014372 | C | 1 |
| AB014374 | C | 1 |
| AB014376 | C | 1 |
| AB014378 | C | 1 |
| AB014379 | C | 1 |
| AB014380 | C | 1 |
| AB014381 | C | 1 |
| AB014382 | C | 1 |
| AB014384 | C | 1 |
| AB014385 | C | 1 |
| AB014386 | C | 1 |
| AB014387 | C | 1 |
| AB014388 | C | 1 |
| AB014389 | C | 1 |
| AB014390 | C | 1 |
| AB014391 | C | 1 |
| AB014392 | C | 1 |
| AB014393 | C | 1 |
| AB014394 | C | 1 |
| AB014395 | C | 1 |
| AB014396 | C | 1 |
| AB014397 | C | 1 |
| AB014398 | C | 1 |
| AB014399 | C | 1 |
| AB048704 | C | 1 |
| AB241112 | C | 1 |
| AB241113 | C | 1 |
| GQ475339 | C | 1 |
| GQ475340 | C | 1 |
| GQ475341 | C | 1 |
| GQ475342 | C | 1 |
| GQ475343 | C | 1 |
| GQ475344 | C | 1 |
| GQ475345 | C | 1 |
| GQ475346 | C | 1 |
| GQ475347 | C | 1 |
| GQ475348 | C | 1 |
| GQ475349 | C | 1 |
| GQ475350 | C | 1 |
| GQ475351 | C | 1 |
| GQ475352 | C | 1 |
| GQ475353 | C | 1 |
| GQ475354 | C | 1 |
| GQ475355 | C | 1 |
| GQ475356 | C | 1 |
| GQ475357 | C | 1 |
| JN418529 | C | 1 |
| JN418530 | C | 1 |
| JN418531 | C | 1 |
| JN418532 | C | 1 |
| JN418533 | C | 1 |
| JN418534 | C | 1 |
| JN418535 | C | 1 |
| JN418536 | C | 1 |
| JN418537 | C | 1 |
| JN418538 | C | 1 |
| JN418539 | C | 1 |
| JN418540 | C | 1 |
| JN418541 | C | 1 |
| JN418542 | C | 1 |
| JN418543 | C | 1 |
| JN418544 | C | 1 |
| JN418546 | C | 1 |
| JN418547 | C | 1 |
| JN418548 | C | 1 |
| JN418549 | C | 1 |
| JN418550 | C | 1 |
| JN418551 | C | 1 |
| JN418552 | C | 1 |
| JN418553 | C | 1 |
| JN418554 | C | 1 |
| JN418555 | C | 1 |
| JN418556 | C | 1 |
| JN418557 | C | 1 |
| JN418558 | C | 1 |
| JN418559 | C | 1 |
| JN418560 | C | 1 |
| JN418561 | C | 1 |
| JN418562 | C | 1 |
| JN418563 | C | 1 |
| JN418564 | C | 1 |
| JN418565 | C | 1 |
| JN418566 | C | 1 |
| JN418567 | C | 1 |
| AY641559 | C | 1 |
| AY641562 | C | 1 |
| AY641563 | C | 1 |
| DQ890381 | C | 1 |
| AB670237 | C | 1 |
| AB670238 | C | 1 |
| AB670239 | C | 1 |
| AB670240 | C | 1 |
| AB670241 | C | 1 |
| AB670242 | C | 1 |
| AB670243 | C | 1 |
| AB670244 | C | 1 |
| AB670245 | C | 1 |
| AB670246 | C | 1 |
| AB670247 | C | 1 |
| AB670249 | C | 1 |
| AB670250 | C | 1 |
| AB670252 | C | 1 |
| AB670253 | C | 1 |
| AB670254 | C | 1 |
| AB670255 | C | 1 |
| AB670256 | C | 1 |
| AB670257 | C | 1 |
| AB670258 | C | 1 |
| AB670259 | C | 1 |
| AB670260 | C | 1 |
| AB670261 | C | 1 |
| AB670262 | C | 1 |
| AB670263 | C | 1 |
| AB670264 | C | 1 |
| AB670265 | C | 1 |
| AB670266 | C | 1 |
| AB670267 | C | 1 |
| AB670268 | C | 1 |
| AB670269 | C | 1 |
| AB670270 | C | 1 |
| AB670271 | C | 1 |
| AB670272 | C | 1 |
| AB670273 | C | 1 |
| AY206374 | C | 1 |
| AY206376 | C | 1 |
| AY206378 | C | 1 |
| AY206379 | C | 1 |
| AY206381 | C | 1 |
| AY206382 | C | 1 |
| AY206384 | C | 1 |
| AY206385 | C | 1 |
| AY206386 | C | 1 |
| AY206388 | C | 1 |
| AY206389 | C | 1 |
| AY206392 | C | 1 |
| FJ904423 | C | 0 |
| AB090270 | D | 0 |
| AB109475 | D | 0 |
| AB109476 | D | 0 |
| AB109477 | D | 0 |
| AB210820 | D | 0 |
| AB222709 | D | 0 |
| AB222710 | D | 0 |
| AB222711 | D | 0 |
| GU456635 | D | 0 |
| GU456636 | D | 0 |
| GU456637 | D | 0 |
| GU456638 | D | 0 |
| GU456639 | D | 0 |
| GU456640 | D | 0 |
| GU456641 | D | 0 |
| GU456642 | D | 0 |
| GU456643 | D | 0 |
| GU456644 | D | 0 |
| GU456645 | D | 0 |
| GU456646 | D | 0 |
| GU456647 | D | 0 |
| GU456648 | D | 0 |
| GU456649 | D | 0 |
| GU456650 | D | 0 |
| GU456651 | D | 0 |
| GU456652 | D | 0 |
| GU456653 | D | 0 |
| GU456654 | D | 0 |
| GU456655 | D | 0 |
| GU456656 | D | 0 |
| GU456657 | D | 0 |
| GU456658 | D | 0 |
| GU456659 | D | 0 |
| GU456660 | D | 0 |
| GU456661 | D | 0 |
| GU456662 | D | 0 |
| GU456663 | D | 0 |
| GU456664 | D | 0 |
| GU456665 | D | 0 |
| GU456666 | D | 0 |
| GU456667 | D | 0 |
| GU456668 | D | 0 |
| GU456669 | D | 0 |
| GU456670 | D | 0 |
| GU456671 | D | 0 |
| GU456672 | D | 0 |
| GU456673 | D | 0 |
| GU456674 | D | 0 |
| GU456675 | D | 0 |
| GU456676 | D | 0 |
| GU456677 | D | 0 |
| GU456678 | D | 0 |
| GU456679 | D | 0 |
| GU456680 | D | 0 |
| GU456681 | D | 0 |
| GU456682 | D | 0 |
| GU456683 | D | 0 |
| GU456684 | D | 0 |
| JF754616 | D | 0 |
| JF754617 | D | 0 |
| JF754618 | D | 0 |
| JF754619 | D | 0 |
| JF754620 | D | 0 |
| JF754621 | D | 0 |
| JF754622 | D | 0 |
| JF754623 | D | 0 |
| JF754624 | D | 0 |
| JF754625 | D | 0 |
| JF754626 | D | 0 |
| JF754627 | D | 0 |
| JF754628 | D | 0 |
| JF754629 | D | 0 |
| JF754630 | D | 0 |
| JF754631 | D | 0 |
| JF754632 | D | 0 |
| JF754633 | D | 0 |
| JF754634 | D | 0 |
| JF754635 | D | 0 |
| AY233291 | D | 0 |
| AY233293 | D | 0 |
| AY233294 | D | 0 |
| AY233295 | D | 0 |
| AY233296 | D | 0 |
| FJ904422 | D | 0 |
| EU155893 | D | 0 |
| AB109478 | D | 0 |
| AB109479 | D | 0 |
| AB110075 | D | 0 |
| AB119251 | D | 0 |
| AB119252 | D | 0 |
| AB119253 | D | 0 |
| AB119254 | D | 0 |
| AB119255 | D | 0 |
| AB119256 | D | 0 |
| JN040769 | D | 0 |
| JN040788 | D | 0 |
| JN040810 | D | 0 |
| JN040817 | D | 0 |
| JN664909 | D | 0 |
| JN664910 | D | 0 |
| JN664911 | D | 0 |
| JN664912 | D | 0 |
| JN664913 | D | 0 |
| JN664914 | D | 0 |
| JN664915 | D | 0 |
| JN664916 | D | 0 |
| JN664917 | D | 0 |
| JN664918 | D | 0 |
| JN664919 | D | 0 |
| JN664920 | D | 0 |
| JN664921 | D | 0 |
| JN664922 | D | 0 |
| AB048702 | D | 0 |
| AB048703 | D | 0 |
| AB090268 | D | 0 |
| AB090269 | D | 0 |
| AB210822 | D | 0 |
| JN040755 | D | 0 |
| JN040777 | D | 0 |
| JN040779 | D | 0 |
| JN040798 | D | 0 |
| JN040800 | D | 0 |
| JN040802 | D | 0 |
| JN040816 | D | 0 |
| JN040820 | D | 0 |
| JN664923 | D | 0 |
| JN664924 | D | 0 |
| JN664925 | D | 0 |
| JN664926 | D | 0 |
| JN664927 | D | 0 |
| JN664928 | D | 0 |
| JN664929 | D | 0 |
| JN664930 | D | 0 |
| JN664931 | D | 0 |
| JN664932 | D | 0 |
| JN664933 | D | 0 |
| JN664934 | D | 0 |
| JN664935 | D | 0 |
| FJ349205 | D | 0 |
| FJ349206 | D | 0 |
| FJ349207 | D | 0 |
| FJ349208 | D | 0 |
| FJ349209 | D | 0 |
| FJ349212 | D | 0 |
| FJ349213 | D | 0 |
| FJ349214 | D | 0 |
| FJ349216 | D | 0 |
| FJ349217 | D | 0 |
| FJ349218 | D | 0 |
| FJ349219 | D | 0 |
| FJ349220 | D | 0 |
| FJ349221 | D | 0 |
| FJ349234 | D | 0 |
| FJ349235 | D | 0 |
| AY373430 | D | 0 |
| AY373431 | D | 0 |
| AY161150 | D | 0 |
| AY161161 | D | 0 |
| JN040750 | D | 0 |
| JN040751 | D | 0 |
| JN040753 | D | 0 |
| JN040754 | D | 0 |
| JN040760 | D | 0 |
| JN040766 | D | 0 |
| JN040772 | D | 0 |
| JN040775 | D | 0 |
| JN040776 | D | 0 |
| JN040786 | D | 0 |
| JN040787 | D | 0 |
| JN040801 | D | 0 |
| JN040804 | D | 0 |
| JN040815 | D | 0 |
| JN664936 | D | 0 |
| JN664937 | D | 0 |
| JN664938 | D | 0 |
| JN664939 | D | 0 |
| JN664940 | D | 0 |
| JN664941 | D | 0 |
| JN664942 | D | 0 |
| JN664944 | D | 0 |
| JN664945 | D | 0 |
| JN664946 | D | 0 |
| JN664947 | D | 0 |
| JN664948 | D | 0 |
| JQ687530 | D | 0 |
| AY161157 | D | 0 |
| AY161159 | D | 0 |
| JQ687531 | D | 0 |
| JQ687532 | D | 0 |
| FJ562263 | D | 0 |
| FJ562309 | D | 0 |
| FJ562338 | D | 0 |
| JF754586 | D | 0 |
| JF754587 | D | 0 |
| JF754589 | D | 0 |
| JF754590 | D | 0 |
| JF754591 | D | 0 |
| JF754592 | D | 0 |
| JF754593 | D | 0 |
| JF754594 | D | 0 |
| JF754595 | D | 0 |
| JF754596 | D | 0 |
| JF754597 | D | 0 |
| JF754598 | D | 0 |
| JF754599 | D | 0 |
| JF754600 | D | 0 |
| JF754601 | D | 0 |
| JF754602 | D | 0 |
| JF754603 | D | 0 |
| JF754604 | D | 0 |
| JF754605 | D | 0 |
| JF754606 | D | 0 |
| JF754607 | D | 0 |
| JF754608 | D | 0 |
| JF754609 | D | 0 |
| JF754610 | D | 0 |
| JF754611 | D | 0 |
| JF754612 | D | 0 |
| JF754613 | D | 0 |
| JF754614 | D | 0 |
| JF754615 | D | 0 |
| FJ904399 | D | 0 |
| FJ904402 | D | 0 |
| FJ904412 | D | 0 |
| FJ904415 | D | 0 |
| FJ904418 | D | 0 |
| FJ904420 | D | 0 |
| FJ904424 | D | 0 |
| FJ904426 | D | 0 |
| FJ904427 | D | 0 |
| FJ904429 | D | 0 |
| FJ904431 | D | 0 |
| FJ904432 | D | 0 |
| FJ904443 | D | 0 |
| FJ904445 | D | 0 |
| FJ904446 | D | 0 |
| FJ904394 | D | 0 |
| FJ904395 | D | 0 |
| FJ904396 | D | 0 |
| FJ904397 | D | 0 |
| FJ904398 | D | 0 |
| FJ904400 | D | 0 |
| FJ904401 | D | 0 |
| FJ904403 | D | 0 |
| FJ904404 | D | 0 |
| FJ904405 | D | 0 |
| FJ904406 | D | 0 |
| FJ904407 | D | 0 |
| FJ904408 | D | 0 |
| FJ904409 | D | 0 |
| FJ904413 | D | 0 |
| FJ904414 | D | 0 |
| FJ904416 | D | 0 |
| FJ904419 | D | 0 |
| FJ904425 | D | 0 |
| FJ904428 | D | 0 |
| FJ904430 | D | 0 |
| FJ904433 | D | 0 |
| FJ904435 | D | 0 |
| FJ904436 | D | 0 |
| FJ904437 | D | 0 |
| FJ904438 | D | 0 |
| FJ904439 | D | 0 |
| FJ904440 | D | 0 |
| FJ904441 | D | 0 |
| FJ904442 | D | 0 |
| FJ904444 | D | 0 |
| FJ904447 | D | 0 |
| AB194947 | E | 0 |
| AB194948 | E | 0 |
| AB205188 | E | 0 |
| AB205189 | E | 0 |
| AB205190 | E | 0 |
| AB205191 | E | 0 |
| AB205192 | E | 0 |
| HM363565 | E | 0 |
| HM363566 | E | 0 |
| HM363567 | E | 0 |
| HM363568 | E | 0 |
| HM363569 | E | 0 |
| HM363570 | E | 0 |
| HM363571 | E | 0 |
| HM363572 | E | 0 |
| HM363573 | E | 0 |
| HM363574 | E | 0 |
| HM363575 | E | 0 |
| HM363576 | E | 0 |
| HM363577 | E | 0 |
| HM363578 | E | 0 |
| HM363579 | E | 0 |
| HM363580 | E | 0 |
| HM363581 | E | 0 |
| HM363582 | E | 0 |
| HM363583 | E | 0 |
| HM363584 | E | 0 |
| HM363585 | E | 0 |
| HM363586 | E | 0 |
| HM363587 | E | 0 |
| HM363588 | E | 0 |
| HM363589 | E | 0 |
| HM363590 | E | 0 |
| HM363591 | E | 0 |
| HM363592 | E | 0 |
| HM363593 | E | 0 |
| HM363594 | E | 0 |
| HM363595 | E | 0 |
| HM363596 | E | 0 |
| HM363597 | E | 0 |
| HM363598 | E | 0 |
| HM363599 | E | 0 |
| HM363600 | E | 0 |
| HM363601 | E | 0 |
| HM363602 | E | 0 |
| HM363603 | E | 0 |
| HM363604 | E | 0 |
| HM363605 | E | 0 |
| HM363606 | E | 0 |
| HM363607 | E | 0 |
| HM363608 | E | 0 |
| HM363609 | E | 0 |
| HM363610 | E | 0 |
| HM363611 | E | 0 |
| FN594748 | E | 0 |
| FN594749 | E | 0 |
| FN594750 | E | 0 |
| FN594751 | E | 0 |
| FN594752 | E | 0 |
| FN594753 | E | 0 |
| FN594755 | E | 0 |
| FN594756 | E | 0 |
| FN594757 | E | 0 |
| FN594758 | E | 0 |
| FN594759 | E | 0 |
| FN594760 | E | 0 |
| FN594761 | E | 0 |
| FN594762 | E | 0 |
| FN594763 | E | 0 |
| FN594764 | E | 0 |
| FN594765 | E | 0 |
| FN594766 | E | 0 |
| GQ161755 | E | 0 |
| GQ161756 | E | 0 |
| GQ161757 | E | 0 |
| GQ161758 | E | 0 |
| GQ161759 | E | 0 |
| GQ161760 | E | 0 |
| GQ161761 | E | 0 |
| GQ161762 | E | 0 |
| GQ161763 | E | 0 |
| GQ161764 | E | 0 |
| GQ161765 | E | 0 |
| GQ161766 | E | 0 |
| GQ161768 | E | 0 |
| GQ161769 | E | 0 |
| GQ161770 | E | 0 |
| GQ161771 | E | 0 |
| GQ161772 | E | 0 |
| GQ161773 | E | 0 |
| GQ161774 | E | 0 |
| GQ161776 | E | 0 |
| GQ161777 | E | 0 |
| GQ161778 | E | 0 |
| GQ161779 | E | 0 |
| GQ161780 | E | 0 |
| GQ161781 | E | 0 |
| GQ161782 | E | 0 |
| GQ161783 | E | 0 |
| GQ161784 | E | 0 |
| GQ161785 | E | 0 |
| GQ161786 | E | 0 |
| GQ161787 | E | 0 |
| GQ161789 | E | 0 |
| GQ161790 | E | 0 |
| GQ161791 | E | 0 |
| GQ161792 | E | 0 |
| GQ161793 | E | 0 |
| GQ161794 | E | 0 |
| GQ161795 | E | 0 |
| GQ161796 | E | 0 |
| GQ161797 | E | 0 |
| GQ161798 | E | 0 |
| GQ161799 | E | 0 |
| GQ161800 | E | 0 |
| GQ161801 | E | 0 |
| GQ161802 | E | 0 |
| GQ161803 | E | 0 |
| GQ161804 | E | 0 |
| GQ161805 | E | 0 |
| GQ161807 | E | 0 |
| GQ161808 | E | 0 |
| GQ161809 | E | 0 |
| GQ161810 | E | 0 |
| GQ161811 | E | 0 |
| GQ161812 | E | 0 |
| GQ161814 | E | 0 |
| GQ161815 | E | 0 |
| GQ161816 | E | 0 |
| GQ161817 | E | 0 |
| GQ161818 | E | 0 |
| GQ161819 | E | 0 |
| GQ161820 | E | 0 |
| GQ161821 | E | 0 |
| GQ161822 | E | 0 |
| GQ161823 | E | 0 |
| GQ161824 | E | 0 |
| GQ161825 | E | 0 |
| GQ161826 | E | 0 |
| GQ161827 | E | 0 |
| GQ161828 | E | 0 |
| GQ161829 | E | 0 |
| GQ161830 | E | 0 |
| GQ161831 | E | 0 |
| GQ161832 | E | 0 |
| GQ161833 | E | 0 |
| GQ161834 | E | 0 |
| GQ161835 | E | 0 |
| GQ161836 | E | 0 |
| EU239217 | E | 0 |
| EU239219 | E | 0 |
| EU239220 | E | 0 |
| EU239221 | E | 0 |
| EU239222 | E | 0 |
| EU239223 | E | 0 |
| EU239224 | E | 0 |
| EU239225 | E | 0 |
| EU239226 | E | 0 |
| FJ349227 | E | 0 |
| AB365446 | F | 0 |
| AB365447 | F | 0 |
| AB365448 | F | 0 |
| AB365449 | F | 0 |
| AB365450 | F | 0 |
| HQ378247 | F | 0 |
| HM585186 | F | 0 |
| HM585187 | F | 0 |
| HM585188 | F | 0 |
| HM585189 | F | 0 |
| HM585190 | F | 0 |
| HM585191 | F | 0 |
| HM585192 | F | 0 |
| HM585193 | F | 0 |
| HM585194 | F | 0 |
| HM585195 | F | 0 |
| HM585196 | F | 0 |
| HM585197 | F | 0 |
| HM585198 | F | 0 |
| HM585199 | F | 0 |
| HM585200 | F | 0 |
| HM590471 | F | 0 |
| HM590472 | F | 0 |
| HM590473 | F | 0 |
| HM590474 | F | 0 |
| HM622135 | F | 0 |
| HM627320 | F | 0 |
| AB205010 | H | 0 |
| AB298362 | H | 0 |
| GU357844 | I | 0 |
| AB486012 | J | 1 |
