# Supplementary material for: The Characteristic Changes in Hepatitis B Virus X Region for Hepatocellular Carcinoma: A Comprehensive Analysis Based on Global Data
Source: PLoS One. 2015 May 5;10(5):e0125555. doi: 10.1371/journal.pone.0125555 (PMC4420286; doi:10.1371/journal.pone.0125555)
Supplement: S2 Table — (DOC) [file pone.0125555.s002.doc]

**S2 Table. Fifty seven references with PUBMED ID or DOI number.**

| NO. | Publications | PUBMED ID or DOI number* |
| --- | --- | --- |
| 1 | J. Gen. Virol. 69, 2575-2583 (1988) | 3171552 |
| 2 | J. Gen. Virol. 67, 2305-2314(1986) | 3783127 |
| 3 | Fukuoka Acta Med. 85 (11), 314-322 (1994) | 7851832 |
| 4 | Arch. Virol. 143 (12), 2313-2326 (1998) | 9930189 |
| 5 | J. Gen. Virol. 82, 883-892 (2001) | 11257194 |
| 6 | Hepatology. 2002 May;35(5):1215-24 | 11981772 |
| 7 | J. Gen. Virol. 83 (PT 11), 2773-2777 (2002) | 12388813 |
| 8 | Gastroenterology. 2003 Jan;124(1):80-90. | 12512032 |
| 9 | J. Med. Virol. 71 (4), 499-503 (2003) | 14556261 |
| 10 | J. Med. Virol. 72, 551-557 (2004) | 14981757 |
| 11 | J. Gen. Virol. 85 (PT 5), 1211-1220 (2004) | 15105537 |
| 12 | CHINESE JOURNAL OF ONCOLOGY, 2004, 26(4):213-6 | 15312382 |
| 13 | Gastroenterology. 2004 Nov;127(5):1356-71 | 15521005 |
| 14 | J. Med. Virol. 75 (1), 13-19 (2005) | 15543574 |
| 15 | J. Infect. Dis. 191 (12), 2022-2032 (2005) | 15897987 |
| 16 | J. Gen. Virol. 86(Pt 6), 1687-1693(2005) | 15914846 |
| 17 | J. Gen. Virol. 86 (PT 7), 2047-2056 (2005) | 15958684 |
| 18 | Jpn. J. Infect. Dis. 58, 244-246 (2005) | 16116261 |
| 19 | J. Med. Virol. 78, 44-52 (2006) | 16299716 |
| 20 | J. Med. Virol. 78, 178-184 (2006) | 16372296 |
| 21 | J. Med. Virol. 78, 341-352 (2006) | 16419116 |
| 22 | J. Gen. Virol. 87 (PT 7), 1873-1882 (2006) | 16760389 |
| 23 | J. Hepatol. 45 (6), 805-812 (2006) | 17050029 |
| 24 | Intervirology 50, 150-155 (2007) | 17191017 |
| 25 | J. Med. Virol. 79 (3), 212-219 (2007) | 17245716 |
| 26 | J. Clin. Virol. 39 (2), 149-152 (2007) | 17442615 |
| 27 | J. Med. Virol. 79, 911-918 (2007) | 17516520 |
| 28 | Intervirology 50 (4), 273-280 (2007) | 17570929 |
| 29 | Asian Pac J Allergy Immunol. 2007 Jun-Sep;25(2-3):183-8. | 18035807 |
| 30 | J. Med. Virol. 80, 217-224 (2008) | 18098129 |
| 31 | J. Gen. Virol. 89 (PT 2), 409-418 (2008) | 18198371 |
| 32 | Virus Res. 132, 174-180 (2008) | 18207274 |
| 33 | J. Med. Virol. 80 (8), 1344-1349 (2008) | 18551607 |
| 34 | Biochem. Biophys. Res. Commun. 374 (4), 773-776 (2008) | 18675784 |
| 35 | J. Gen. Virol. 90 (PT 7), 1622-1628 (2009) | 19339480 |
| 36 | J. Med. Virol. 81 (6), 983-987 (2009) | 19382274 |
| 37 | J. Gen. Virol. 90 (PT 10), 2442-2451 (2009) | 19535503 |
| 38 | J. Virol. 83 (20), 10538-10547 (2009) | 19640977 |
| 39 | J. Med. Virol. 82 (3), 379-389 (2010) | 20087936 |
| 40 | J. Gen. Virol. 91(Pt 6), 1609-1620(2010). | 20147517 |
| 41 | PLoS ONE 5 (2), E9297 (2010) | 20174575 |
| 42 | Biochem. Biophys. Res. Commun. 394 (3), 574-580 (2010) | 20214885 |
| 43 | J. Med. Virol. 82 (7), 1126-1134 (2010) | 20513074 |
| 44 | PLoS ONE 5 (7), E11615 (2010) | 20657838 |
| 45 | J. Gastroenterol. 46 (3), 391-400 (2011) | 20848146 |
| 46 | J. Med. Virol. 83 (4), 594-601 (2011) | 21328372 |
| 47 | J. Med. Virol. 83 (6), 948-952 (2011) | 21503905 |
| 48 | J. Med. Virol. 83 (8), 1321-1325 (2011) | 21678435 |
| 49 | Mem. Inst. Oswaldo Cruz 106 (4), 495-498 (2011) | 21739039 |
| 50 | J. Med. Virol. 83 (9), 1530-1536 (2011) | 21739442 |
| 51 | J. Clin. Virol. 52 (2), 151-154 (2011) | 21802353 |
| 52 | J. Med. Virol. 83 (10), 1717-1726 (2011) | 21837787 |
| 53 | BMC Cancer. 2011 Oct 21;11:458. | 22014121 |
| 54 | J. Med. Virol. 84 (3), 414-423 (2012) | 22246826 |
| 55 | J. Immunol. 189 (1), 279-286 (2012) | 22661095 |
| 56 | Clin. Microbiol. Infect. 18 (10), E412-E418 (2012) | 22827722 |
| 57 | Arch Biol Sci 64, 1319-1326 (2012) | DOI:10.2298/ABS1204319L* |
| *Publication only afforded DOI information. | |  |
