# Supplementary material for: The Characteristic Changes in Hepatitis B Virus X Region for Hepatocellular Carcinoma: A Comprehensive Analysis Based on Global Data
Source: PLoS One. 2015 May 5;10(5):e0125555. doi: 10.1371/journal.pone.0125555 (PMC4420286; doi:10.1371/journal.pone.0125555)
Supplement: S3 Table — (DOC) [file pone.0125555.s003.doc]

**S3 Table. Amino acid differences between HCC and Non-HCC patients with HBV genotype C infection.**

| AA location | Predominant residue (%) | | Main mutations | | | Mutant ratio in groups (%) | | *P* value^a^ | *P* value^b^ | OR |
| --- | --- | --- | --- | --- | --- | --- | --- | --- | --- | --- |
| HBx | Non-HCC | HCC |  | | | Non-HCC | HCC | (Univariate) | (Multivariate) | 95% CI |
| 5 | Val | Val | Leu | → | Met | 13.7% | 20.8% | 0.03 | 0.962 | / |
|  | 65.6% | 67.4% |  |  |  |  |  |  |  |  |
| 6 | Cys | Cys | Cys | → | Arg/Tyr | 0.0% | 2.1% | 0.02 | 0.404 | / |
|  | 100.0% | 97.9% |  |  |  |  |  |  |  |  |
| 29 | Pro | Pro | Ser | → | Pro | 96.6% | 100.0% | 0.02 | 0.178 | / |
|  | 96.6% | 100.0% |  |  |  |  |  |  |  |  |
| 36 | Thr | Pro | Ala/Thr | → | Pro | 25.3% | 45.8% | < 0.001 | 0.001 | 2.37 |
|  | 52.5% | 45.8% |  |  |  |  |  |  |  | 1.41-4.01 |
| 36 |  |  |  | → | Ser | 7.8% | 15.3% | < 0.001 | < 0.001 | 3.7 |
|  |  |  |  |  |  |  |  |  |  | 1.87-7.33 |
| 38 | Pro | Pro | Pro | → | Ser | 17.6% | 29.9% | 0.01 | 0.004 | 2.15 |
|  | 81.7% | 70.1% |  |  |  |  |  |  |  | 1.27-3.63 |
| 94 | His | His | His | → | Tyr | 18.9% | 35.4% | < 0.001 | < 0.001 | 2.74 |
|  | 79.8% | 64.6% |  |  |  |  |  |  |  | 1.72-4.39 |
| 106 | Thr | Thr | Thr | → | Ile/Ser | 0.0% | 4.2% | 0.02 | 0.21 | / |
|  | 99.0% | 95.8% |  |  |  |  |  |  |  |  |
| 116 | Leu | Leu | Val | → | Leu | 58.4% | 82.6% | < 0.001 | 0.003 | 2.37 |
|  | 58.4% | 82.6% |  |  |  |  |  |  |  | 1.35-4.16 |
| 143 | Cys | Cys | Cys | → | Arg | 0.3% | 3.5% | 0.01 | 0.006 | 23.47 |
|  | 99.5% | 96.5% |  |  |  |  |  |  |  | 2.45-225.26 |
| ^a^ Fisher's exact test; ^b^ Stepwise Forward (Conditional) logistic regression | | | | | | | | | | |
