# Supplementary material for: The Characteristic Changes in Hepatitis B Virus X Region for Hepatocellular Carcinoma: A Comprehensive Analysis Based on Global Data
Source: PLoS One. 2015 May 5;10(5):e0125555. doi: 10.1371/journal.pone.0125555 (PMC4420286; doi:10.1371/journal.pone.0125555)
Supplement: S4 Table — (DOC) [file pone.0125555.s004.doc]

**S4 Table. Distribution of genotype C HCC risk mutations among four Asian countries.**

| Genotype C HCC risky mutations | 1383C | | 1479C | | 1479T | | 1485T | | 1631T | | 1653T | | 1719T | | 1800C | |
| --- | --- | --- | --- | --- | --- | --- | --- | --- | --- | --- | --- | --- | --- | --- | --- | --- |
| Japan (n=106) | 53 | 50.0% | 55 | 51.9% | 22 | 20.8% | 33 | 31.1% | 8 | 7.5% | 46 | 43.4% | 89 | 84.0% | 5 | 4.7% |
| Mainland China (n=12) | 3 | 25.0% | 3 | 25.0% | 0 | 0.0% | 3 | 25.0% | 0 | 0.0% | 1 | 8.3% | 7 | 58.3% | 0 | 0.0% |
| Phillippines (n=2) | 2 | 100.0% | 0 | 0.0% | 0 | 0.0% | 2 | 100.0% | 0 | 0.0% | 1 | 50.0% | 2 | 100.0% | 0 | 0.0% |
| South Korea (n=23) | 18 | 78.3% | 13 | 56.5% | 0 | 0.0% | 5 | 21.7% | 4 | 17.4% | 3 | 13.0% | 20 | 87.0% | 0 | 0.0% |
| P value* | **0.002** |  | **<0.001** |  |  |  | 0.186 |  | 0.152 |  | **0.003** |  | 0.147 |  | 0.75 |  |
| *Fisher's exact test was used. *P* < 0.05 was considered significant and marked in bold. | | | | | | | | | | |  |  |  |  |  |  |
