# Supplementary material for: The Characteristic Changes in Hepatitis B Virus X Region for Hepatocellular Carcinoma: A Comprehensive Analysis Based on Global Data
Source: PLoS One. 2015 May 5;10(5):e0125555. doi: 10.1371/journal.pone.0125555 (PMC4420286; doi:10.1371/journal.pone.0125555)
Supplement: S5 Table — (DOC) [file pone.0125555.s005.doc]

| **S5 Table. Distribution of genotype C HCC risk amino acid residues across different HBV genotypes in HCC patients.** | | | | | | | | | | |
| --- | --- | --- | --- | --- | --- | --- | --- | --- | --- | --- |
| Amino acid | aa36 | | aa38 | | aa94 | | aa116 | | aa143 | |
| position | Predominant residue (%) | Gt C HCC risky residue | Predominant residue (%) | Gt C HCC risky residue | Predominant residue (%) | Gt C HCC risky residue | Predominant residue (%) | Gt C HCC risky residue | Predominant residue (%) | Gt C HCC risky residue |
|  |  | Pro/Ser |  | Ser |  | Tyr |  | Leu |  | Arg |
| Gt C HCC (n=144) | Pro (45.8%) | 45.8%/15.3% | Pro (70.1%) | 29.9% | His (64.6%) | 35.4% | Leu (82.6%) | 82.6% | Cys (96.5%) | 3.5% |
| Gt A HCC (n=2) | Ala/Thr (50%/50%) | 0 | Ser (100%) | 100% | His (100%) | 0.0% | Val (100%) | 0.0% | Cys (100%) | 0.0% |
| Gt B HCC (n=14) | Ala (92.9%) | Ser (7.1%) | Pro (100%) | 0.0% | His (100%) | 0.0% | Val (100%) | 0.0% | Cys (85.7%) | 14.3% |
| Gt J HCC (n=1) | Pro (100%) | Pro (100%) | Pro (100%) | 0.0% | His (100%) | 0.0% | Val (100%) | 0.0% | Cys (100%) | 0.0% |
| *P* value* |  | **<0.001** |  | **0.002** |  | **0.006** |  | **<0.001** |  | 0.231 |
| *Fisher's exact test. aa, amino acid. Gt, genotype. *P* < 0.05 was considered significant and shown in bold. | | | | | | | | | | |
