# Supplementary material for: The Characteristic Changes in Hepatitis B Virus X Region for Hepatocellular Carcinoma: A Comprehensive Analysis Based on Global Data
Source: PLoS One. 2015 May 5;10(5):e0125555. doi: 10.1371/journal.pone.0125555 (PMC4420286; doi:10.1371/journal.pone.0125555)
Supplement: S6 Table — (DOC) [file pone.0125555.s006.doc]

| **S6 Table. Distribution of genotype C HCC risk amino acid residues across AsC with different HBV genotypes.** | | | | | | | | | | |
| --- | --- | --- | --- | --- | --- | --- | --- | --- | --- | --- |
| Position | aa36 | | aa38 | | aa94 | | aa116 | | aa143 | |
|  | Predominant residue (%) | Gt C HCC risky residue | Predominant residue (%) | Gt C HCC risky residue | Predominant residue (%) | Gt C HCC risky residue | Predominant residue (%) | Gt C HCC risky residue | Predominant residue (%) | Gt C HCC risky residue |
|  |  | Pro/Ser |  | Ser |  | Tyr |  | Leu |  | Arg |
| Gt A AsC (n=20) | Thr (80%) | 0.0% | Ser (100%) | 100.0% | His (95%) | 5.0% | Val (95%) | 5.0% | Cys (100%) | 0.0% |
| Gt B AsC (n=3) | Ala (100%) | 0.0% | Pro (100%) | 0.0% | His (100%) | 0.0% | Val (100%) | 0.0% | Cys (66.7%) | 33.3% |
| Gt C AsC (n=18) | Pro (38.9%)/Thr (38.9%) | Pro (38.9%) | Pro (77.8%) | 22.2% | His (83.3%) | 16.7% | Leu (72.2%) | 72.2% | Cys (100%) | 0.0% |
| Gt D AsC (n=161) | Thr (72%) | Pro (0.6%)/Ser (3.7%) | Ser (98.8%) | 98.8% | His (95.7%) | 3.7% | Leu (98.8%) | 98.8% | Cys (100%) | 0.0% |
| Gt E AsC (n=158) | Asp (84.2%) | 0.0% | Ser (95.6%) | 95.6% | His (91.8%) | 8.2% | Leu (99.4%) | 99.4% | Cys (100%) | 0.0% |
| Gt F AsC (n=5) | Ala (100%) | 0.0% | Pro (100%) | 0.0% | His (60%) | 20.0% | Val (100%) | 0.0% | Cys (100%) | 0.0% |
| Gt I AsC (n=1) | Asp (100%) | 0.0% | Ser (100%) | 100.0% | His (100%) | 0.0% | Val (100%) | 0.0% | Cys (100%) | 0.0% |
| *P* value* |  | **<0.001** |  | **<0.001** |  | **0.029** |  | **<0.001** |  | **0.01** |
| *Fisher's exact test. aa, amino acid. Gt, genotype. *P* < 0.05 was considered significant and shown in bold. | | | | | | | | | | |
